# Copepod Population-Specific Response to a Toxic Diatom Diet

**DOI:** 10.1371/journal.pone.0047262

**Published:** 2012-10-08

**Authors:** Chiara Lauritano, Ylenia Carotenuto, Antonio Miralto, Gabriele Procaccini, Adrianna Ianora

**Affiliations:** Stazione Zoologica Anton Dohrn, Villa Comunale, Napoli, Italy; University of Hamburg, Germany

## Abstract

Diatoms are key phytoplankton organisms and one of the main primary producers in aquatic ecosystems. However, many diatom species produce a series of secondary metabolites, collectively termed oxylipins, that disrupt development in the offspring of grazers, such as copepods, that feed on these unicellular algae. We hypothesized that different populations of copepods may deal differently with the same oxylipin-producing diatom diet. Here we provide comparative studies of expression level analyses of selected genes of interest for three *Calanus helgolandicus* populations (North Sea, Atlantic Ocean and Mediterranean Sea) exposed to the same strain of the oxylipin-producing diatom *Skeletonema marinoi* using as control algae the flagellate *Rhodomonas baltica*. Expression levels of detoxification enzymes and stress proteins (e.g. glutathione S-transferase, glutathione synthase, superoxide dismutase, catalase, aldehyde dehydrogenases and heat shock proteins) and proteins involved in apoptosis regulation and cell cycle progression were analyzed in copepods after both 24 and 48 hours of feeding on the diatom or on a control diet. Strong differences occurred among copepod populations, with the Mediterranean population of *C. helgolandicus* being more susceptible to the toxic diet compared to the others. This study opens new perspectives for understanding copepod population-specific responses to diatom toxins and may help in underpinning the cellular mechanisms underlying copepod toxicity during diatom blooms.

## Introduction

Diatoms are key phytoplankton organisms in the world’s oceans and are considered essential in the transfer of energy through marine food chains. However, in the last 20 years, numerous studies have shown that these unicellular plants at times produce secondary metabolites with toxic effects on reproduction and development of marine organisms such as crustacean copepods [1], [2], [3] and cladocerans [4], echinoderm sea urchins [5] and sea stars [6], [7], polychaete worms [8], [9], and ascidians [10]. Diatom metabolites are the end-products of a lipoxygenase/hydroperoxide lyase metabolic pathway [11], [12], [13], [14], [15] initiated by damage to algal cells, as occurs through grazing by predators. Cell damage activates lipase enzymes, which liberate polyunsaturated fatty acids (PUFAs) from cell membranes that are immediately oxidized and cleaved within seconds to form polyunsaturated aldehydes (PUAs) and a plethora of other metabolites collectively termed oxylipins.

Oxylipins, and PUAs in particular, can compromise embryonic and larval development in marine organisms by inhibiting fertilization processes, reducing larval fitness and inducing malformations in the offspring of grazers that feed on these unicellular algae (as reviewed by [16]). Teratogenesis and reduction in egg production and hatching success have been observed also for wild copepods feeding on the natural winter/spring diatom-dominated bloom in the Mediterranean Sea (i.e. Adriatic Sea [1], [2]), in North and South Pacific (i.e. Dabob Bay, Washington, USA [17] and the coastal zone off Dichato, Chile [18]) and in the Baltic Sea [19]. Such antiproliferative compounds may impact on herbivory by sabotaging future generations of grazers, thereby allowing diatom blooms to persist when grazing pressure would otherwise have caused them to crash.

In recent studies [20], [21], we showed that expression levels of selected genes of interest (GOI) were significantly reduced when females of the copepod *Calanus helgolandicus* (*C. helgolandicus*) were fed for two days (d) on the diatom *Skeletonema marinoi* (*S. marinoi*) which is known to produce high quantities of PUAs and several other oxylipins including fatty acid hydroperoxides, hydroxyl- and keto-fatty acids, and epoxyalcohols [3]. On the contrary, a diet of *Chaetoceros socialis*, which does not produce any aldehydes, but only low levels of other oxylipins, did not induce significant expression levels changes [21]. Interestingly, after 2 d of feeding on *S. marinoi* egg viability in *C. helgolandicus* was still high (90%), and decreased rapidly to 10% after 5 d ([3], supporting material), indicating that changes in gene expression levels after 2 d could act as an early warning signal to denote a deterioration in copepod fitness.

The aim of the present study was to further explore the toxic effects of ingestion of *S. marinoi* on gene expression levels in three different *C. helgolandicus* populations: Swedish western coast (Gullmar Fjord, North Sea), English Channel (NE Atlantic Ocean) and North Adriatic Sea (Mediterranean Sea) populations. The three populations are exposed to different diatom blooms in terms of species composition [2], [22], [23], [24]. All three *Calanus* populations co-exist with *S. marinoi*; in the Swedish west coast *S. marinoi* is differentiated in local populations and represents the most abundant diatom species reaching peak abundances almost twice a year [24]; in the NE Atlantic this diatom occurs commonly, but it is never the most abundant diatom species, and is replaced by other oxylipin-producing diatoms (*Rhizosolenia delicatula*, *Thalassiosira rotula*, *Chaetoceros sp*., etc.; [25]); in the North Adriatic Sea *S. marinoi* is the most abundant species during the winter-spring phytoplankton bloom [2]. PUAs have been detected in all three sampling sites [25], [26], [27].

We analyzed expression levels of selected GOI in the three *Calanus* populations after 24 and 48 h of feeding on the stationary phase of growth of the same toxic *S. marinoi* clone used by Gerecht et al. [28]. The clone was isolated from the diatom bloom in the North Adriatic Sea in 1997 and was shown to produce 2.1 [SD 1.1] fmol PUAs cell^−1^ and 1.1 [SD 0.8] fmol cell^−1^ of nonvolatile oxylipins, such as hydroxy acids and epoxy alcohols, in the stationary phase of growth, more than 10 years after it was first isolated. PUA production measurements in other cultivated *S. marinoi* strains indicated PUA concentration between 0.1 and 25 fmol cell^−1^, depending on the nutrient status of the diatom cells, while higher concentrations have been found at sea (up to 47.7 fmol cell^−1^), probably due to optimum growth conditions of the field algal population [27], [29], [30]. Considering ingestion rates of about 1,000 diatom cells per hour [1], in our experiments *C. helgolandicus* had ingested 76.8 and 153.6 pmol of oxylipins after 24 h and 48 h, respectively.

The selected genes were utilized in previous studies on *C. helgolandicus* response to toxic diatom diets [21] and are known to have a primary role in generic stress responses, defense systems (e.g. aldehyde, free fatty acid and free radical detoxification) or apoptosis regulation in other organisms, from humans to marine organisms [31], [32], [33], [34], [35]. In particular, we analyzed the heat shock protein families 40 and 70 (HSP40 and HSP70, respectively) activated in response to various environmental stress factors [36], the microsomal cytochrome P450 family 4 monooxygenases (CYP4) involved in oxidative modification (known as Phase I reaction) of chemicals into more hydrophilic metabolites to enhance their elimination or inactivation [37], catalase (CAT) and superoxide dismutase (SOD) [38] responsible for detoxification of reactive oxygen species (ROS) [3], and the antioxidant activity of glutathione synthase (GSH-S) and glutathione S-transferase (GST). Six aldehyde dehydrogenase (ALDH) isoforms (ALDH2, ALDH3, ALDH6, ALDH7, ALDH8 and ALDH9) involved in aldehyde detoxification due to lipid peroxidation (LPO) [35], [39] were examined because of their possible role in PUA detoxification. Finally, we analyzed 3 apoptosis-regulating genes, an inhibitor of the apoptosis protein (IAP), the cell cycle and apoptosis regulatory 1 protein (CARP), the cellular apoptosis susceptibility protein (CAS), and the microtubule subunits (alpha and beta tubulins) for their involvement in apoptosis regulation and cell-cycle progression [40], [41], [42].

## Results

### Population Identification

A 518 bp fragment of the mitochondrial Cytochrome Oxidase subunit I region (COI) was amplified for each *C. helgolandicus* population. COI sequence in animals collected from the Atlantic Ocean corresponded to the haplotype H1 typical of individuals of the NE Atlantic (GenBank accession number AY942600) as published by Papadopoulos and co-workers [43]. COI sequence in animals collected from the Adriatic Sea corresponded to the haplotype H8 (GenBank accession number AY942593) associated with specimens living in the NE Atlantic and/or Adriatic Sea. COI sequence in animals collected from the Swedish western coast corresponded to H1, H17 (GenBank accession number AY942591), H18 (GenBank accession number AY942595), HQ150067 or new haplotypes (GenBank accession number JX070087 and JX070088). COI sequences differed only for a maximum of 3 out of 518 nucleotides between the three populations. These nucleotide substitutions did not induce amino-acid changes.

### Expression Level of Genes of Interest (GOI)

#### Swedish calanus helgolandicus population

**Figure 1:**
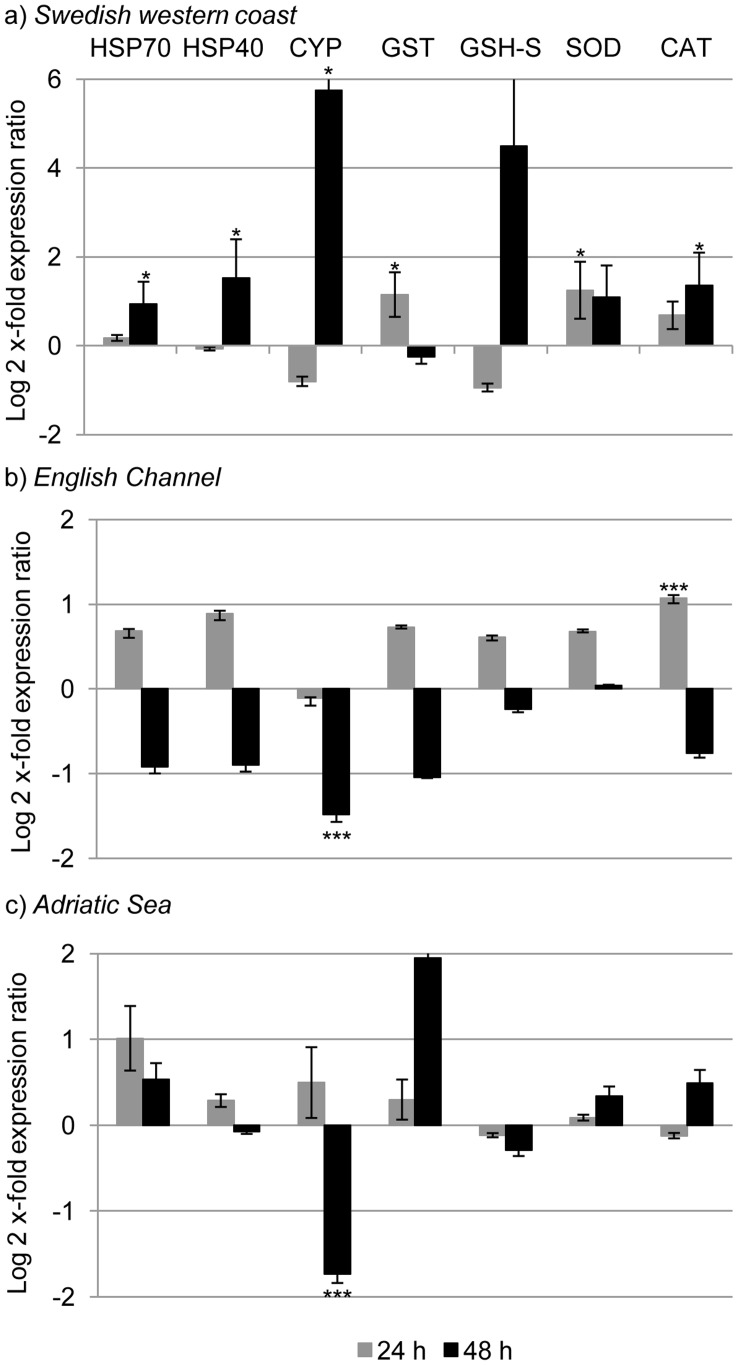
Expression levels of genes involved in stress and defense systems in the copepod *Calanus helgolandicus*. Changes in expression levels of Heat shock protein 70 (HSP70) and 40 (HSP40), Cytochrome P450-4 (CYP4), Glutathione S-Transferase (GST), Glutathione Synthase (GSH-S), Catalase (CAT) and Superoxide Dismutase (SOD) genes in Swedish (a), English Channel (b) and Adriatic (c) *C. helgolandicus* specimens fed *Skeletonema marinoi* (*S. marinoi*) for 24 or 48 h compared to expression levels in females fed on the control *Rhodomonas baltica* (represented in the figure by x-axis). The ribosomal protein S20 was used as reference gene to normalize the data.

**Figure 2:**
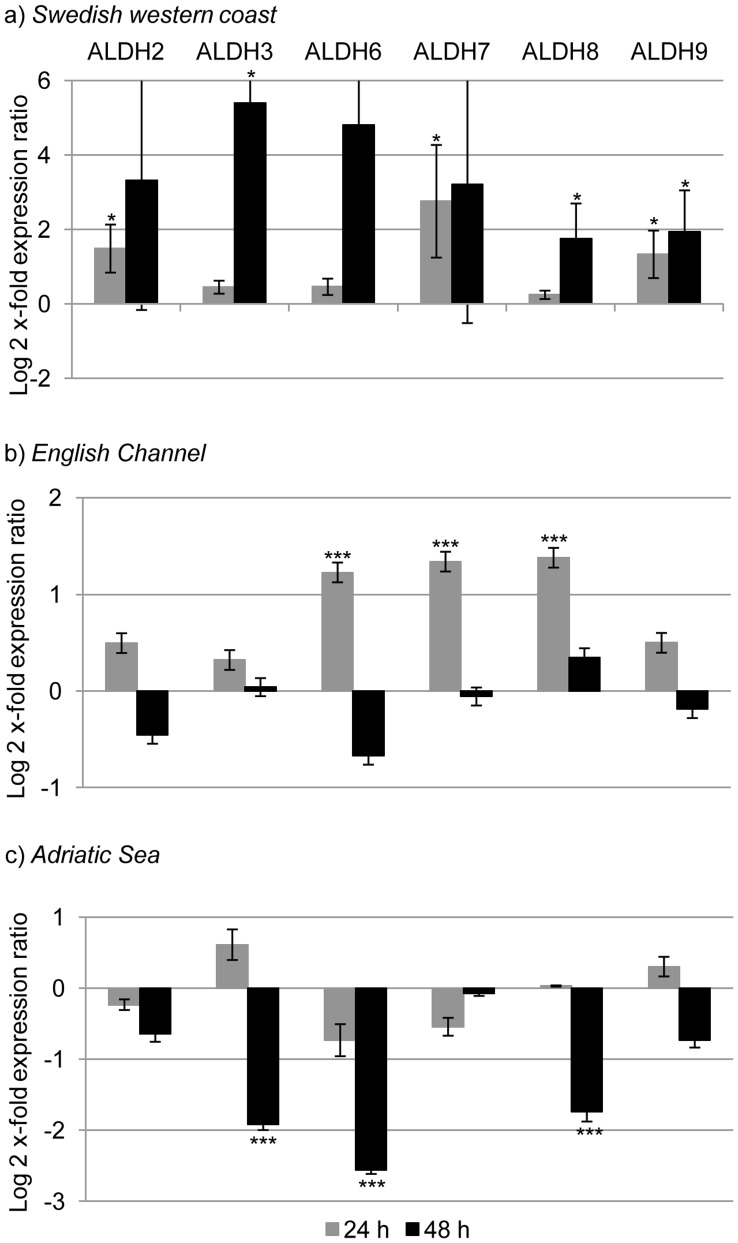
Relative gene expression levels of aldehyde dehydrogenases (ALDH) in the copepod *Calanus helgolandicu*s. Changes in ALDH2, ALDH3, ALDH6, ALDH7, ALDH8 and ALDH9 gene expression levels in Swedish (a), English Channel (b) and Adriatic (c) *C. helgolandicus* females fed *Skeletonema marinoi* (*S. marinoi*) for 24 or 48 h compared to expression levels in females fed on the control *Rhodomonas baltica* (represented in the figure by x-axis). The ribosomal protein S20 was used as reference gene to normalize the data.

**Figure 3:**
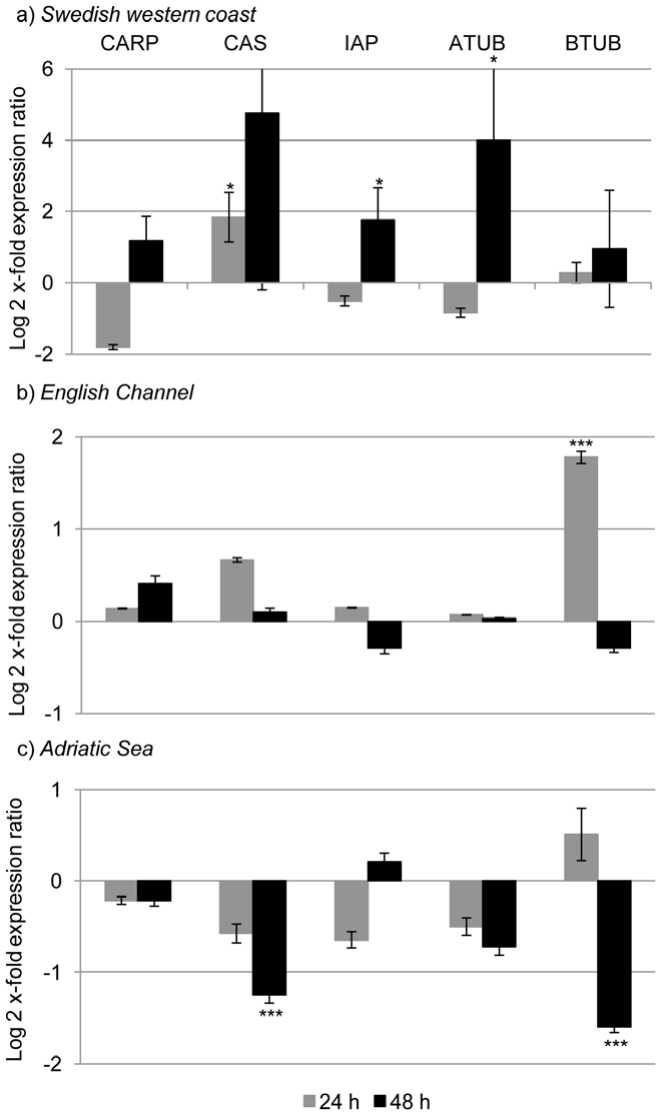
Expression analysis of genes involved in apoptosis and mitotic spindle formation in *Calanus helgolandicus*. Changes in expression levels of Cell Cycle and Apoptosis Regulatory 1 Protein (CARP), Cellular Apoptosis Susceptibility Protein (CAS), Inhibitor of Apoptosis Protein (IAP), and Alpha and Beta tubulins (ATUB and BTUB) genes in Swedish (a), English Channel (b) and Adriatic (c) *C. helgolandicus* fed *Skeletonema marinoi* (*S. marinoi*) for 24 or 48 h compared to expression levels in females fed on the control *Rhodomonas baltica* (represented in the figure by x-axis). The ribosomal protein S20 was used as reference gene to normalize the data.

After 24 h of feeding on *S. marinoi*, both primary defense and aldehyde detoxification genes increased. GST, SOD, ALDH2, ALDH7, ALDH9 and CAS expression levels were significantly up-regulated (p value<0.05 for all the genes; Figures 1a, 2a and 3a). After 48 h, there was a stronger response and many GOI were up-regulated (HSP70, HSP40, CYP, CAT, ALDH3, ALDH8, ALDH9, IAP and ATUB; p value<0.05 for all the genes) (Figures 1a, 2a and 3a).

#### English channel calanus helgolandicus population

After 24 h, CAT was up-regulated (p value<0.001) (Figure 1b) and there was also the activation of three out of six genes involved in the aldehyde detoxification complex: ALDH6, ALDH7 and ALDH8 were up-regulated (p value<0.001 for all the genes) (Figure 2b). BTUB expression levels significantly increased after 24 h of ingestion of *S. marinoi* (p value<0.001) (Figure 3b). After 48 h, no significant changes were observed, except for a significant reduction of CYP expression levels (p value<0.001) (Figure 1b).

#### Adriatic sea calanus helgolandicus population

Expression levels of genes belonging to primary defense system, or aldehyde detoxification and apoptosis regulation did not show significant changes in *C. helgolandicus* specimens fed *S. marinoi* for 24 h. After 48 h, many GOI showed a pattern of down-regulation: CYP, ALDH3, ALDH6, ALDH8, CAS, BTUB (p value< 0.001 for all the genes) (Figures 1c, 2c and 3c).

## Discussion

Our results provide new insight on the often debated toxic/non-toxic effects of diatoms on copepod reproduction and development in laboratory and field studies [1], [2], [44], [45]. Of the three *Calanus helgolandicus* populations tested, the Swedish population seems to be better capable of coping with the toxic *S. marinoi* diet by activating almost all stress/detoxification proteins after 24 and 48 h. The Atlantic population only activated the free radical detoxification enzyme CAT and some aldehyde dehydrogenases soon after stress exposure (24 h), but not after prolonged exposure. On the contrary, the Adriatic population was unable to activate defense enzymes after both 24 and 48 h and showed a general pattern of down-regulation after 48 h of diatom exposure, as shown in previous experiments [21]. Thus it appeared to be the most sensitive population to the toxic diet. Accordingly, strong reduction in egg production and hatching success have been found for Adriatic *C. helgolandicus* fed on *S. marinoi* in laboratory experiments or during natural diatom blooms in the North Adriatic Sea [1], [2], but these effects were not observed for Atlantic copepods [25], [44]. Hence, gene expression patterns observed in this study may correlate with copepod physiological responses.

Analyses of population genetic diversity of Mediterranean and Atlantic *C. helgolandicus* populations suggest Pleistocene divergence between the two basins and species vicariance [43]. Our data concord with previous findings of low distinction between *C. helgolandicus* mtDNA haplotypes (genetic divergences between 0.22% and 0.57%, [43]), which is lower than interspecific divergence (17% to 22% between *Calanus finmarchicus*, *C. helgolandicus* and *Calanus glacialis*, [46]; 7–25% between ten *Calanus* species [47]). The Adriatic population does not seem to be connected by gene flow with the other two populations, since there is no common haplotype in the Mediterranean and extra-Mediterranean samples. The Atlantic and the North Sea populations, instead, share a single haplotype. Interestingly, despite the minor genetic differences among populations of *C. helgolandicus*, large physiological differences in tolerance to toxic oxylipins were present.

In recent years, numerous studies have focused on the effects of stressors on aquatic organisms, showing that responses to toxicants tend to be species-specific and may also be due to pre-adaptation to a given xenobiotic [48], [49], [50], [51]. For example, pre-exposure of the aquatic oligochaete *Sparganophilus pearsei* to mercury in their native sediments influenced the resistance levels recorded during laboratory mercury exposure [51]. Colin and Dam [48] showed that when two geographically distant populations of the copepod *Acartia hudsonica* were reared on the toxic dinoflagellate *Alexandrium fundyense*, the one that had not experienced recurrent blooms of the toxic algae had lower somatic growth, size at maturity, egg production, and survival, compared to the other population that showed no effects on these life-history parameters. Our results confirmed these studies supporting and implementing them, for the first time, by gene expression studies.

Defense and detoxification proteins, such as heat shock proteins, antioxidant and ROS detoxification enzymes, have been analyzed in copepods exposed to various environmental contaminants, such as heavy metals, endocrine disruptor chemicals and hydrocarbons. The data indicate high inter- and intra-species variability in copepod responses, depending on the type of stressor tested, the concentration and exposure time, and the enzyme isoform studied [50].

In this study, enzymes involved in free radical detoxification were up-regulated after both 24 and 48 h of exposure to a diatom diet in the Swedish population, after 24 h in the Atlantic population, but not in the Mediterranean population of *C. helgolandicus*. These data suggest an immediate specific capability of the Swedish and Atlantic populations to protect themselves against radical toxicants. A simultaneous increase in both SOD and CAT has also been observed in *C. finmarchicus* after 12 h of exposure to low naphthalene concentrations [52]. Thereafter levels for both antioxidants returned to basal levels, except after 48 h, when CAT levels were still elevated in copepods exposed to intermediate naphthalene concentrations. Whereas Hansen and co-workers [52] concluded that there was no clear relationship between antioxidant mRNA levels and exposure time/concentration, our data suggest that antioxidant defense genes (e.g. GST, SOD and CAT) and also a more specific detoxification system (ALDHs) could be activated in the Atlantic and Swedish populations soon after stress exposure (24 h).

In fact, ALDH2, ALDH7 and ALDH9 increased after 24 h of *S. marinoi* exposure, and all the six analyzed ALDHs increased after 48 h in the Swedish population (even if with high variability between replicates for some genes); ALDH6, ALDH7 and ALDH8 increased after 24 h in the Atlantic population; no ALDHs were up-regulated in the Mediterranean population. Since Atlantic and Swedish copepods are more frequently exposed to diatoms [23], [24], [25] they may have evolved mechanisms to better cope with deleterious diatom oxylipins.

After 48 h of *S. marinoi* exposure, HSP40 and HSP70 increased in the Swedish population suggesting a protective chaperoning activity. Romano et al. [53] have also recently shown that sea urchins activate HSP70 when challenged with low concentrations (0.25 mg/ml) of the PUA decadienal thereby protecting embryos against the toxic effects of this aldehyde. This up-regulation was only found at 9 h post fertilization (hpf), whereas at 5, 24 and 48 hpf, expression levels were comparable to the control.

A protective role is also suggested by the activation of the cellular apoptosis susceptibility protein (CAS) and the inhibitor of the apoptosis protein (IAP). IAP proteins are generally known for the control of cell death and the inhibition of apoptosis, however new emerging functions have been attributed to this protein family, such as cytoprotective and cellular stress response functions [54]. In this experiment, IAP expression levels significantly increased in the Swedish population after 48 h of exposure to the toxic diatom, while it showed no significant results for the other populations. CAS is essential for cell survival, can associate with microtubules and mitotic spindles, and is necessary for the mitotic checkpoint that assures the accurate segregation of chromosomes to daughter cells [40], [55]. Supporting the hypothesis of a Swedish population more resistant to diatom toxins compared to the others is the fact that CAS expression levels increased in the Swedish population, and decreased in the Adriatic one (similar to our previous findings [21]). IAP was down-regulated in our previous experiments in Adriatic *C. helgolandicus* specimens fed on *S. marinoi* in the same experimental conditions, but, in the present study, values were comparable to the control, probably due to inter-individual variability.

Our results indicate strong population-level variations in copepod detoxification mechanisms to toxic diatoms and may explain why diatoms at times did not reduce hatching success in previous studies [44], [45]. Such population-specific differences in tolerance to toxic metabolites suggest that co-evolution between diatoms and copepods is also based on a chemical arms race between plant defenses and animal offenses and the evolution of phenotypic traits among populations of a single herbivore species. These results are consistent with the hypothesis of evolved grazer resistance to toxins in copepod populations that have a longer history of exposure to pronounced and long-lasting spring phytoplankton blooms mainly dominated by diatoms such as those that occur in the North Atlantic Ocean [56].

## Materials and Methods

### Copepod Sampling and Feeding Experiments

No specific permits were required for the described field studies, the locations were not privately-owned or protected in any way and the field studies did not involve endangered or protected species.

*Calanus helgolandicus* specimens were collected in three different geographical locations: North Adriatic Sea (Mediterranean Sea), Swedish west coast (Gullmar Fjord) and North Atlantic Ocean. *C. helgolandicus* were collected in the North Adriatic Sea in April 2011, transported to Stazione Zoologica Anton Dohrn (SZN) in Naples and transferred to 10 L tanks. Specimens collected in the North Atlantic Ocean (May 2010) were transported to the CNRS of Roscoff (France) and transferred to 10 L tanks. Specimens collected in the Swedish west coast (May 2011) were transported to the Sven Lovén Centre for Marine Sciences, University of Gothenburg (Kristineberg) and transferred to 10 L tanks. In all the three cases, 120 adult female *C. helgolandicus* were sampled from the tanks under a Leica stereomicroscope and transferred to triplicate 1 L bottles (20 animals/bottle) filled with 0.22 µm filtered sea water (FSW) enriched with either unialgal diets of the control non-oxylipin producing flagellate *Rhodomonas baltica* (*R. baltica*) (7500–8000 cells/ml), that does not impair copepod egg production and hatching success [57], or the toxic oxylipin-producing diatom *Skeletonema marinoi* (*S. marinoi*) (45.000–60.000 cells/ml) provided ad libitum in the stationary phase of growth. Bottles containing copepods were maintained in temperature controlled rooms at 8–18°C (without altering the natural sea water temperature). To avoid settlement of diatom cells to container bottoms, bottles were gently rotated every 4 h. This was not necessary with bottles containing free-swimming flagellate cells.

Both algal strains belong to the SZN culture collection. *R. baltica* (Strain SZN FE202) was cultured in glass jars with 0.22 µm-FSW enriched with k medium at 20°C and on a 12∶12 h dark:light cycle. The diatom *S. marinoi* (Strain SZN FE6) was cultured under the same experimental conditions but with F2 medium. Every day FSW and new food was added to each bottle at the same concentration as the day before. After 24 h and 48 h, triplicate sub-samples of 5 animals for each diet were collected and transferred to FSW for 24 h to eliminate any algal residues in the gut. After this, each replicate was carefully transferred to 500 µl Trizol Reagent (Invitrogen), frozen directly in liquid nitrogen and stored at −80°C until DNA or RNA extraction.

### DNA Extraction and Population Identification

Total DNA was extracted from a pool of 5 animals from the Atlantic Ocean and Adriatic Sea copepod populations and from 22 single animals (22 replicates) from the Swedish population, according to Trizol manufacturer’s protocol (Invitrogen). DNA quantity was assured by Nano-Drop (ND-1000 UV-Vis spectrophotometer; NanoDrop Technologies). In all the three cases, the following primers were used to amplify a 518 bp fragment of the mitochondrial Cytochrome Oxidase subunit I region (COI): ChelgCOI-F (5′-GGCCAAAACAGGGAGAGATA-3′) and ChelgCOI-R (5′-CGGGACTCAGTATAATTATTCGTCTA-3′) [43]. Reactions were carried out in 20 µl volume with 2 µl of 10× PCR reaction buffer Roche, 2 µl of 0.1% BSA, 2 µl of 10× 2 mM dNTP, 0.8 µl of 5 U/µl Taq Roche, 1 µl of 20 pmol/µl for each oligo, 1,5 µl template DNA and nuclease-free water to 20 µl. The PCR program consisted of a denaturation step at 94°C for 3 min, 35 cycles at 94°C for 1 min, 50°C for 45 sec and 72°C for 1 min, and a final extension step at 72°C for 7 min. Amplified PCR products were analyzed by 1.5% agarose gel electrophoresis in TBE buffer. In order to verify the correct assignment of amplicons to COI region, the resulting bands were excised from the gel and extracted according to the QIAquick Gel Extraction Kit protocol (QIAGEN) and sequences analyzed. The identity of each sequence was confirmed using the bioinformatics tool BLAST (Basic local alignment search tool).

### RNA Extraction and cDNA Synthesis

Total RNA was extracted using Trizol manufacturer’s protocol (Invitrogen). RNA quantity and purity was assured by Nano-Drop (ND-1000 UV-Vis spectrophotometer; NanoDrop Technologies), RNA quality by gel electrophoresis. 1 µg of each RNA was retrotranscribed into cDNA with the iScriptTM cDNA Synthesis Kit (BIORAD) following the manufacturer’s instructions, using the GeneAmp PCR System 9700 (Perkin Elmer). The reaction was carried out in 20 µl final volume with 4 µl 5× iScript reaction mix, 1 µl iScript reverse transcriptase and H_2_O. The mix was first incubated 5 min at 25°C, followed by 30 min at 42°C and finally heated at 85°C for 5 min.

### Reverse Transcription-Quantitative Real Time Polymerase Chain Reaction (RT-qPCR)

The fluorescent dye SYBR GREEN was used to evaluate expression levels of the selected genes by RT-qPCR. Fluorescence was monitored once per cycle after product extension and increased above background fluorescence at a cycle number that depended on the initial template concentration. RT-qPCR was performed in MicroAmp Optical 384-Well reaction plate (Applied Biosystem) with Optical Adhesive Covers (Applied Biosystem) in a Viia7 Real Time PCR System (Applied Biosystem). The PCR volume for each sample was 10 µl, with 5 µl of Fast Start SYBR Green Master Mix (Roche), 1 µl of cDNA template and 0.7 pmol/µl for each oligo. The RT-qPCR thermal profile was obtained using the following procedure: 95°C for 10 min, 40 times 95°C for 15 sec and 60°C for 1 min, 72°C for 5 min. The programme was set to reveal the melting curve of each amplicon from 60°C to 95°C, and read every 0.5°C.

All RT-qPCR reactions were carried out in triplicate to capture intra-assay variability. Each assay included three no-template negative controls (NTC) for each primer pair. Reaction efficiencies for all primer pairs have been previously calculated using the equation E = 10^−1/slope^ [21]. Primer’s sequences, efficiencies and correlation coefficients, and gene accession numbers were previously published [21]. A 1∶100 template dilution (4 ± 2 ng) was used which allowed almost all gene amplifications to fit in the optimal read window (from 15 to 25 cycles). Only a single peak in the melting-curve analyses of all genes was identified, confirming a gene-specific amplification and the absence of primer-dimers.

To study expression levels for each target gene relative to the most stable RG, S20 [20], we used the REST tool (Relative expression software tool) [58]. Copepods fed on the dinoflagellate *Rhodomonas baltica*, which does not produce any PUAs and oxylipins, were used as control condition. Statistical analysis was performed using GraphPad Prism version 4.00 for Windows (GraphPad Software, San Diego, California, USA).
